# Zinc oxide nanoparticles produced by *Zingiber officinale* ameliorates acute toxoplasmosis-induced pathological and biochemical alterations and reduced parasite burden in mice model

**DOI:** 10.1371/journal.pntd.0011447

**Published:** 2023-07-06

**Authors:** Asmaa M. El-kady, Abeer S. Hassan, Khalil Mohamed, Mashael S. Alfaifi, Hayam Elshazly, Zaenah Zuhair Alamri, Majed H. Wakid, Hattan S. Gattan, Sarah A. Altwaim, Wafa Abdullah I. Al-Megrin, Salwa Younis

**Affiliations:** 1 Department of Medical Parasitology, Faculty of Medicine, South Valley University, Qena, Egypt; 2 Department of Pharmaceutics, Faculty of pharmacy, South Valley University, Qena, Egypt; 3 Department of Epidemiology, Faculty of Public Health and Health Informatics, Umm Al-Qura University, Mecca, Saudi Arabia; 4 Department of Biology, Faculty of Sciences-Scientific Departments, Qassim University, Buraidah, Qassim, Saudi Arabia; 5 Department of Zoology, Faculty of Science, Beni-Suef University, Beni Suef, Egypt; 6 Department of Biology, College of Science, University of Jeddah, Jeddah, Saudi Arabia; 7 Department of Medical Laboratory Sciences, Faculty of Applied Medical Sciences, King Abdulaziz University, Jeddah, Saudi Arabia; 8 Special Infectious Agents Unit, King Fahd Medical Research Center, King Abdulaziz University, Jeddah, Saudi Arabia; 9 Department of Clinical Microbiology and Immunology, Faculty of Medicine, King Abdulaziz University, Jeddah, Saudi Arabia; 10 Department of Biology, College of Science, Princess Nourah bint Abdulrahman University, Riyadh, Saudi Arabia; 11 Department of Medical Parasitology, Faculty of Medicine, Alexandria University, Alexandria, Egypt; Babol University of Medical Science, ISLAMIC REPUBLIC OF IRAN

## Abstract

**Background:**

Although, approximately 30% of the world’s population is estimated to be infected with *Toxoplasma gondii* (*T*. *gondii*) with serious manifestations in immunocompromised patients and pregnant females, the available treatment options for toxoplasmosis are limited with serious side effects. Therefore, it is of great importance to identify novel potent, well tolerated candidates for treatment of toxoplasmosis. The present study aimed to evaluate the effect of Zinc oxide nanoparticles (ZnO NPs) synthesized using *Zingiber officinale* against acute toxoplasmosis in experimentally infected mice.

**Methods:**

The ethanolic extract of ginger was used to prepare ZnO NPs. The produced ZnO NPs were characterized in terms of structure and morphology using Fourier Transformed Infrared Spectroscopy (FTIR), X-Ray Diffraction (XRD), UV- spectroscopy and scanning electron microscopy (SEM). The prepared formula was used in treatment of *T*. *gondii* RH virulent strain. Forty animals were divided into four groups, with ten mice per group. The first group was the uninfected, control group. The second group was infected but untreated. The third and the fourth groups received ZnO NPs and Spiramycin orally in a dose of 10 mg/kg and 200 mg/kg/day respectively. The effect of the used formulas on the animals survival rate, parasite burden, liver enzymes -including Alanine transaminase (ALT) and aspartate transaminase (AST)-, nitric oxide (NO) and Catalase antioxidant enzyme (CAT) activity was measured. Moreover, the effect of treatment on histopathological alterations associated with toxoplasmosis was examined.

**Results:**

Mice treated with ZnO NPs showed the longest survival time with significant reduction in the parasite load in the livers and peritoneal fluids of the same group. Moreover, ZnO NPs treatment was associated with a significant reduction in the level of liver enzymes (ALT, AST) and NO and a significant increase in the antioxidant activity of CAT enzyme. SEM examination of tachyzoites from the peritoneal fluid showed marked distortion of *T*. *gondii* tachyzoites isolated from mice treated with ZnO NPs in comparison to untreated group. *T*. *gondii* induced histopathological alterations in the liver and brain were reversed by ZnO NPs treatment with restoration of normal tissue morphology.

**Conclusion:**

The produced formula showed a good therapeutic potential in treatment of murine toxoplasmosis as demonstrated by prolonged survival rate, reduced parasite burden, improved *T*. *gondii* associated liver injury and histopathological alterations. Thus, we assume that the protective effect observed in the current research is attributed to the antioxidant capability of NPs. Based on the results obtained from the current work, we suggest greenly produced ZnO NPs as a chemotherapeutic agent with good therapeutic potential and high levels of safety in the treatment of toxoplasmosis.

## Author summary

A considerable fraction of the human and animal population worldwide is affected by toxoplasmosis, which is caused by the common apicomplexan parasite *T*. *gondii*. Toxoplasmosis affects more than 30% of the world’s population, which is a serious public health hazard. There are several ways to catch the disease, including eating undercooked meat containing tissue cysts, consuming food or water that contaminated with mature oocytes, and congenitally by passing from a pregnant woman to her own fetus. Those who have undergone organ or blood transfusions from infected people may also contract it. In immunocompetent humans, toxoplasmosis typically results in minimal symptoms, while it can cause minor clinical signs such enlarged lymph nodes and flu-like symptoms. Toxoplasmosis can cause a wide range of symptoms of varying severity in people with a weakened immune system, such as cancer patients, people with HIV/AIDS, and people who have received organ transplants. If left untreated, these symptoms can include encephalitis, seizures, vision problems, poor coordination, and even death. Moreover, toxoplasmosis may result in miscarriage in women who contract the illness during pregnancy or neuropsychological symptoms in neonates who have the virus at birth, such as hydrocephalus, blindness, mental retardation, encephalitis, etc. Unfortunately, therapeutic options for toxoplasmosis are limited by the serious side effects of the drugs (including severe toxicity, protracted treatment, high cost, and potential parasite resistance). Consequently, it is crucial for researchers to discover new anti-*Toxoplasma* medications with targeted activity that could get rid of tissue cysts as well as tachyzoites. In the present study we used ZnO NPs prepared using ginger extract in treatment of *T*. *gondii* RH virulent strain in experimentally infected mice. The produced formula showed a good therapeutic potential in treatment of murine toxoplasmosis as demonstrated by prolonged survival rate, reduced parasite burden, improved *T*. *gondii* associated liver injury and histopathological alterations. Thus, we assume that the protective effect observed in the current research is attributed to the antioxidant capability of NPs. Our results indicated greenly produced ZnO NPs as a novel anti-*Toxoplasma* agent with high levels of safety in the treatment of the disease.

## Introduction

*Toxoplasma gondii* (*T*. *gondii*) is a protozoan intracellular parasite that infects almost all animals along with humans [1,2]. Estimates indicated that around 30% of the world’s population is infected with *T*. *gondii* [2]. Intermediate hosts- including humans- can get infected via ingesting raw meat containing *T*. *gondii* tissue cysts (bradyzoites) or water and food contaminated with oocysts excreted by cats [3].

Thirty-four outbreaks of clinical toxoplasmosis in humans have been documented throughout the past 50 years [4]. In immunocompetent people, symptoms are minimal and mimic the flu [4–6]. On the other hand, toxoplasmosis is considered as a serious condition among those with compromised immune systems [2]. In both HIV-positive and non-HIV immunocompromised individuals, the most frequent clinical symptom caused by *T*. *gondii* infection is toxoplasmic encephalitis (TE) [2]. Pneumonia, retinochoroiditis, and disseminated systemic manifestations can also be recognized [7]. Despite treatment, the forementioned conditions are life-threatening with mortality ranges from 38% to 67% [8]. Therefore, chemotherapeutics for toxoplasmosis should be highly effective and cross the BBB (blood brain barrier).

There are limited options for treatment of toxoplasmosis. Trimethoprim (TMP) and pyrimethamine (PYR) are the main drugs used for treatment of acute toxoplasmosis [9]. They have to be used in combination regimens with sulfonamides since they give unsatisfactory outcome when used alone. These treatment modalities have serious side effects including myelotoxic side effects that necessitate therapeutic cessation or result in patient noncompliance [9]. This is a significant drawback because *T*. *gondii*-infected patients frequently need lengthy courses of treatment. In addition to poor patient compliance and various adverse events, drug resistance in *T*. *gondii* is regarded as a minor problem. From congenitally infected neonates in Brazil, Silva et al. recently obtained a sulfadiazine-resistant *T*. *gondii* strain [10].

Although the combination of pyrimethamine and sulphadiazine, is the main line of treatment of toxoplasmosis, it is not advised for the treatment of pregnant females with acute toxoplasmosis [11]. Spiramycin is an alternate medication used in this situation [12]. It is a well-known macrolide that has long been demonstrated to be effective against mouse *Toxoplasma* infection [12]. In humans, the use of Spiramycin therapy resulted in a 60% reduction in the overall risk of foetal transmission following maternal *Toxoplasma* infection during pregnancy [13]. The major disadvantage of the drug is being having an inhibitory rather than a curative impact [14]. Therefore, it is necessary to find new, effective therapeutic agents that can act on both tachyzoites and cysts with a high level of safety for all patients’ categories including pregnant women and newborns.

In recent years, major advances in the development of innovative pharmaceutical delivery methods have been made thanks to the rising notion of nanotechnology in medical science research. Recent efforts in this area could pave the way for the development of novel pharmacological formulations with distinguishing features that improve therapeutic effectiveness and patient compliance. A growing number of nano-formulations is becoming interested in the inherent qualities of metal and metal oxide nanoparticles [15]. Nowadays, a growing number of research is being conducted on the green, physical, and chemical production of nanoscale metals [16,17]. Green synthesis of nanoparticles uses natural and environmentally-friendly materials as end-capping agents and dispersants at the same time to reduces energy consumption and avoid toxic and harmful reagents [18]. When compared to chemical and physical methods of nanoparticle preparation, green synthesis has many advantages as being non-toxic [18], pollution-free [19], echo-friendly, economical [20], and more sustainable [21].

Zinc oxide nanoparticles (ZnO NPs) have attracted a lot of attention due to their various potential applications in a wide range of industries. ZnO NPs were synthesized using a variety of traditional chemical and physical techniques, although some toxic byproducts could have negative impacts on medicinal applications. Due to their benefits, such as simplicity, eco-friendliness, and reduced level of toxicity when compared to ZnO NPs generated by chemical and physical processes, green synthesis approaches have grown in importance [22,23].

The goal of the current work was the evaluation of ZnO NPs produced using the ethanolic extract of ginger in treatment of animals experimentally infected with *RH* strain of *T*. *gondii*. The efficacy of the formula in comparison to the reference drug (Spiramycin) was evaluated in terms of parasite burden, biochemical tests and histopathological examination of brain and liver of infected animals.

## Materials and methods

### 1. Ethics statement

The present experiment was conducted in the department of Medical Parasitology, Faculty of Medicine, Alexandria University. Animal experiments were carried out in accordance with the guidelines of the Declaration of Helsinki after approval by the Research and Ethics Committee of the Faculty of Medicine, Alexandria University (protocol code**: 0306016**). Animal care followed the National Institute of Health (NIH) Guide for care and use of laboratory animals.

### 2. Preparation of ZnO NPs

**Fig 1:**
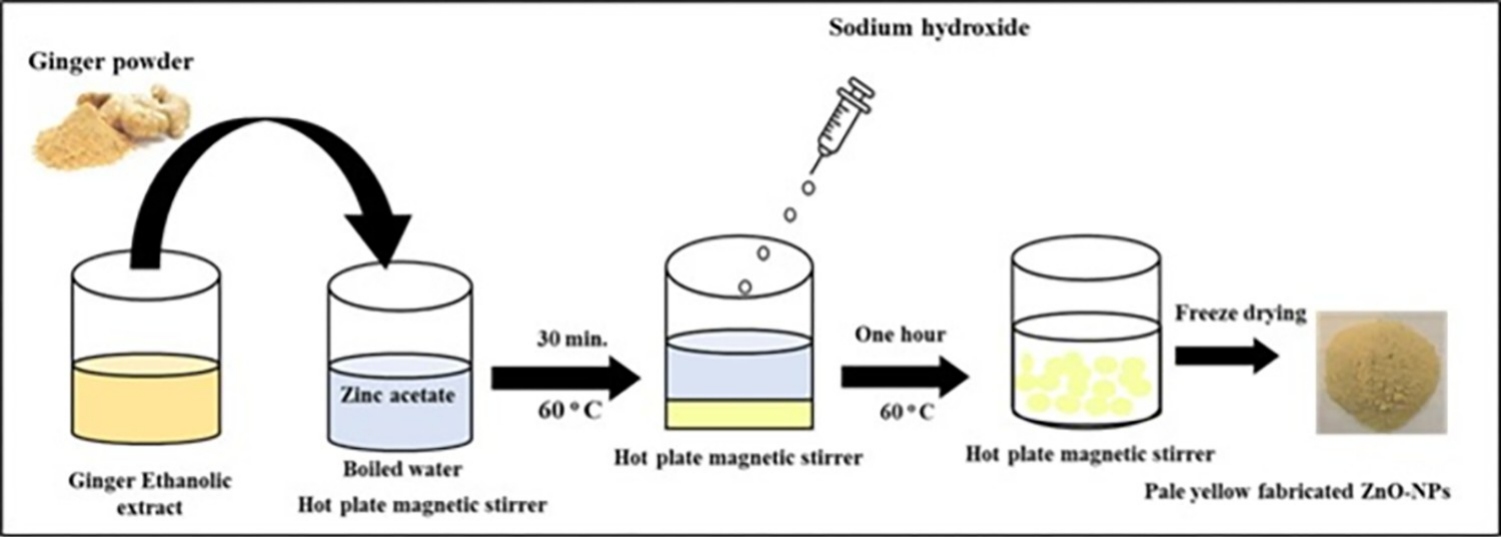
Schematic representation for green synthesis of ZnO NPs using ginger ethanolic extract.

ZnO NPs were produced using the ethanolic extract of ginger following the method presented in previous research [24]. Briefly, dried ginger (0.5g) was dissolved in ethanol (100 mL), then was mixed with 5 g of zinc acetate previously dissolved in boiled distilled water (1000 mL). After that, the mixture was swirled for 30 minutes at 60°C on a hot plate with a magnetic stirrer. The reaction’s pH was then raised to 12 by the addition of Sodium hydroxide, at which point ZnO NPs precipitated. ZnO NPs were completely reduced from zinc acetate in the mixture by stirring for one hour. This was followed by centrifugation of the mixture at 4000 rpm and washing twice with distilled water and once with ethanol. After that, the sediment was dried and freezed resulting in a pale yellow powder as shown in Fig 1.

## 3. Characterization of ZnO NPs

### 3.1. UV–Visible spectrophotometry

The obtained product was examined using a double-beam spectrophotometer (Thermo Scientific Evolution 300 UV–Vis Spectrophotometer; Thermo Fisher Scientific, Waltham, MA, USA) to confirm the formation of ZnO NPs formulation.

### 3.2. Fourier transformed infrared (FTIR) spectroscopy

FTIR analysis was carried out to characterize the prepared nano-formulation using FTIR spectrophotometer (IR-470, Shimadzu, Kyoto, Japan). The obtained sample of ZnO NPs was blended with potassium bromide and were hydraulically pressed into discs. The infrared spectrum was recorded from 4000 to 400 cm^- 1^.

### 3.3. X-Ray diffraction (XRD)

X-ray diffraction analysis was used to characterize ZnO NPs. The XRD patterns of the prepared NPs were recorded using a powder X-ray diffractometer (Bruker D8 discover) operating at 30 kV, 10 mA and equipped with Cu Kα radiation of λ = 1.5406 Å with the angle adjusted in the range of 2θ = 5° to 2θ = 80° and a step size of 0.0505°. The following equation used to calculate the average size of ZnO NPs [25]:

$$D=\frac{K\lambda }{\beta Cos\theta }$$

Where D is the average particle size in nm, λ is the wavelength of the X ray diffraction (0.15406 nm), β is full width at half maximum (FWHM in radians) of the diffraction peak, K is the Scherrer constant (shape factor) with the value of 0.9–1 and θ is the Bragg diffraction angle.

### 3.4. SEM (Scanning Electron Microscopy)

Surface morphology of green synthesized ZnO NPs was studied using SEM (FEI 200 Quanta FEG, FEI, USA) at an acceleration voltage of 20.00 kV. A Thin film of purified ZnO NPs was used. Then, the images of ZnO NPs were captured at 60,000× magnifications.

## 4. Experimental design and animal grouping

Forty male Swiss Albino mice aging four to six weeks old and weighing 20 to 25 g were used. Mice were kept in cages that were properly ventilated, with free access to water, and regular pellet meals. Strict 12-hour light/12-hour dark cycles at 25±2°C were applied to all mouse groups. Stool samples from all mice were examined over the course of three consecutive days in order to rule out any parasite infections [26] [27].

In the present experiment, we used *T*. *gondii* RH virulent strain. The strain was maintained in the laboratory and tachyzoites were isolated from the peritoneal fluid of previously infected mice on the 5^th^ day post-infection. Briefly, peritoneal fluid collected from euthanized infected mice was washed with saline for three times. Then a hemocytometer was used to count the number of tachyzoites and adjust the dose for infection of animals at 2500 tachyzoites/100 μL saline /mouse [28].

Animals under study were distributed into four groups (ten mice each). Three groups were infected with *T*. *gondii* while one group was left unaffected. Infected mice were categorized as following: infected untreated control, infected–ZnO NPs treated and infected-Spiramycin treated. Before treatments, a homogenous suspension of powdered ZnO NPs in ethanol was prepared [29]. It was administered at a dose of 10 mg/ kg/day [30]. On the same line, Spiramycin was given at a dose of 200 mg/kg/day—at the same time daily—after being dissolved in 100 μL of saline [12,31]. Treatments were given orally with an esophageal tube starting at the same day of infection [32–34] for 5 consecutive days [35–37]. After the end of treatments, mice were sacrificed on the 6^th^ day post infection for evaluation of the efficacy of the used drugs.

### Evaluation of the effectiveness of the used formula as anti-*Toxoplasma* Agent

Assessment of each drug efficacy was carried out as follows:

### 4.1. Survival rate

Mice from all experimental groups were monitored daily to create the survival rate using Kaplan–Meier survival curve [38,39].

### 4.2. Determination of parasite count and percent reduction (%R) of parasite load

The number of tachyzoites was counted in the liver and the peritoneal fluid. Livers were isolated from sacrificed mice. Impression smears were prepared from each liver and stained with Giemsa. *T*. *gondii* tachyzoites were counted under light microscope (×400) lens. The mean values for each mouse and for each group were calculated. On the other hand, the peritoneal fluid from experimentally infected mice was collected separately and spun at 2000 rpm for five minutes to determine the number of tachyzoites [35]. The sediment was resuspended in one ml phosphate buffer saline (PBS) (pH 7.4). Extracellular tachyzoites were then counted under light microscope (x400) using Neubauer haemocytometer. The mean tachyzoite count for each mouse and for each group was determined.

The % R in the mean tachyzoites count in the peritoneal fluid and liver was determined using the following equation [40]:

$$\%R=\frac{Mean\;number\;of\;tachyzoites\;in\;control\;group_{i}\;Mean\;number\;of\;tachyzoites\;in\;treated\;group}{Mean\;number\;of\;tachyzoites\;in\;control\;group}\;X\;100$$

### 4.3. Morphological examination of collected tachyzoites by SEM

The peritoneal fluid from all animals was collected on the sacrifice day for examination by SEM. It was immediately fixed in glutaraldehyde and processed for SEM examination of the ultra-structure of the tachyzoites (JSM-IT 200, JOEL, Tokyo, Japan) [39].

### 4.4. Biochemical analysis

*4*.*4*.*1*. *Liver functions*. Blood was collected from euthanized mice and centrifuged for extraction of serum. The levels of Aspartate aminotransferase (AST) and alanine aminotransferase (ALT) in the serum were measured calorimetrically using kits obtained from Randox Laboratories Co. (Crumlin, U.K.) [41].

*4*.*4*.*2*. *Oxidant/antioxidant assessment*. NO (Nitric oxide) is produced by a variety of cells in response to parasitic infections and has been demonstrated to play a significant role in immune protection against several protozoans, including *T*. *gondii* [42]. On the other hand, it is responsible for triggering harmful pathological alterations in the infected hosts [43]. So, we measured serum NO level in the blood of all animal groups using a biodiagnostic assay kits according to Green et al. [43].

On the same context, the antioxidant activity of green synthesized ZnO NPs was evaluated. Catalase antioxidant enzyme (CAT) activity in mice plasma was determined by measuring the conversion of hydrogen peroxide to oxygen with a Clark-type electrode connected to a YSI 5300A Biological Oxygen Monitor (Yellow Springs, OH, USA) [44].

### 4.5. Histopathological study

After the mice were slaughtered, the livers and brains of each study group animals were isolated. Collected tissues were fixed in 10% formalin. Hematoxylin and eosin (H&E) stained sections were then prepared for microscopic examination. Architecture, portal inflammation severity (mild, moderate, and marked), lytic necrosis, apoptosis, and localized inflammation were all evaluated in liver sections. On the other hand, sections of brain tissue were inspected for necrosis, inflammation, hemorrhage, interstitial edema, cells with vacuolated cytoplasm, congested blood vessels, shrunken cells, and interstitial edema.

### 4.6. Statistical analysis of the data

Data were analyzed using IBM SPSS software package version 20.0. (IBM Corp, Armonk, NY, USA). ANOVA was used for comparing animal groups under study, followed by the post hoc test (Tukey) for pairwise comparison. Survival rate curve was created using Kaplan–Meier survival curve. The obtained results were considered significant at p<0.05.

## Results

### 1. Synthesis of ZnO NPs

ZnO NPs were successfully synthesized using ginger ethanolic extract as a reducing agent (Fig **1**). The obtained ZnO NPs were characterized for particle size, crystallin structure and surface morphology.

### 2. Characterization of the developed ZnO NPs

#### 2.1. UV–Visible spectrophotometry examination of the obtained NPs

**Fig 2:**
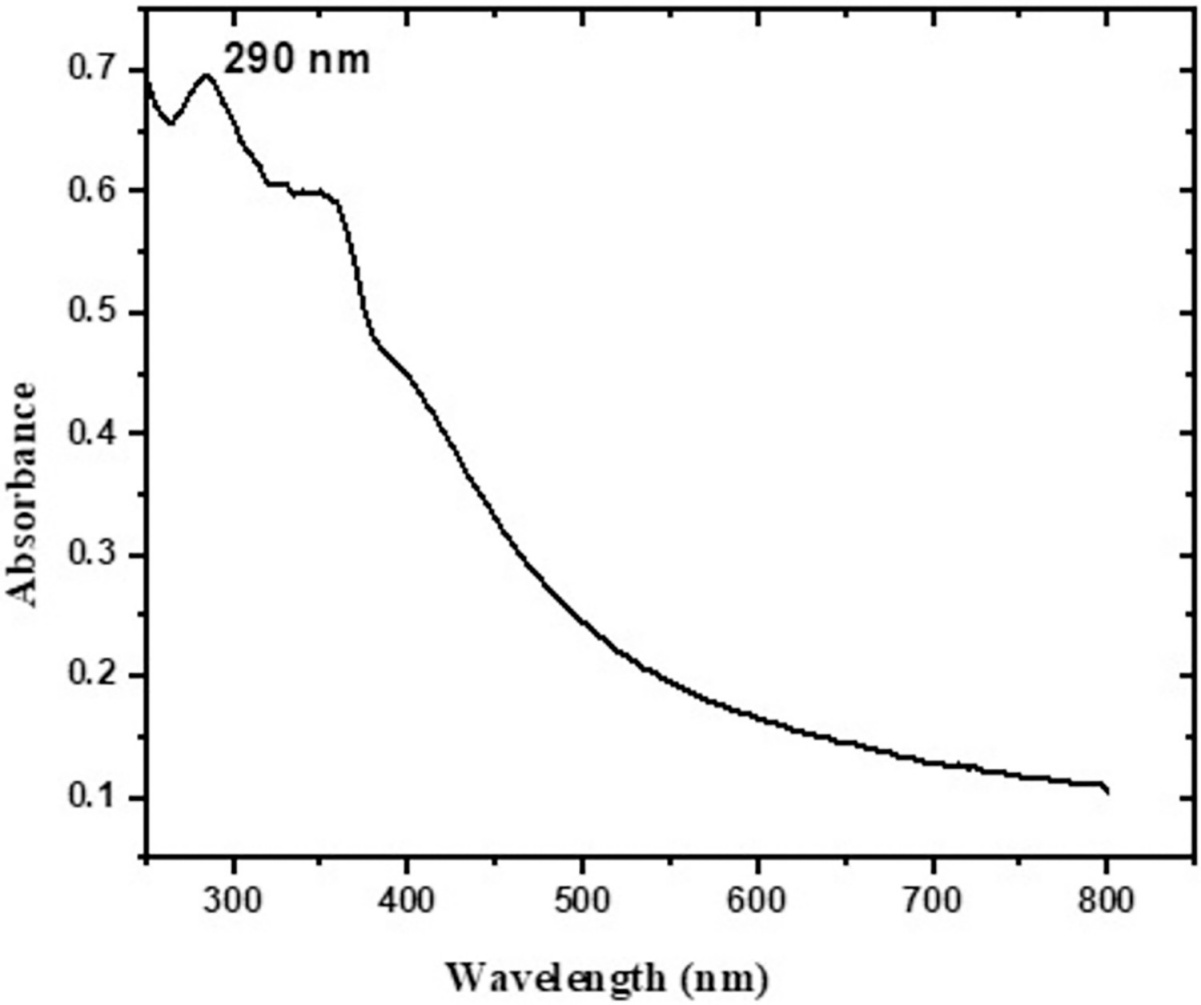
UV–Visible spectrum of ZnO NPs prepared using ginger.

The confirmation of the production of ZnO NPs was done using UV-Vis spectroscopic technique with a wavelength range from 200 to 800 nm. ZnO NPs prepared from ginger extract showed maximum absorbance peak at 290 nm (**Fig 2**). Nanoscale synthesized ZnO NPs exhibit absorbance patterns at shorter wavelength when compared to conventional ZnO.

### 2.2. Fourier transform infrared spectroscopy (FTIR) analysis of the prepared ZnO NPs

**Fig 3:**
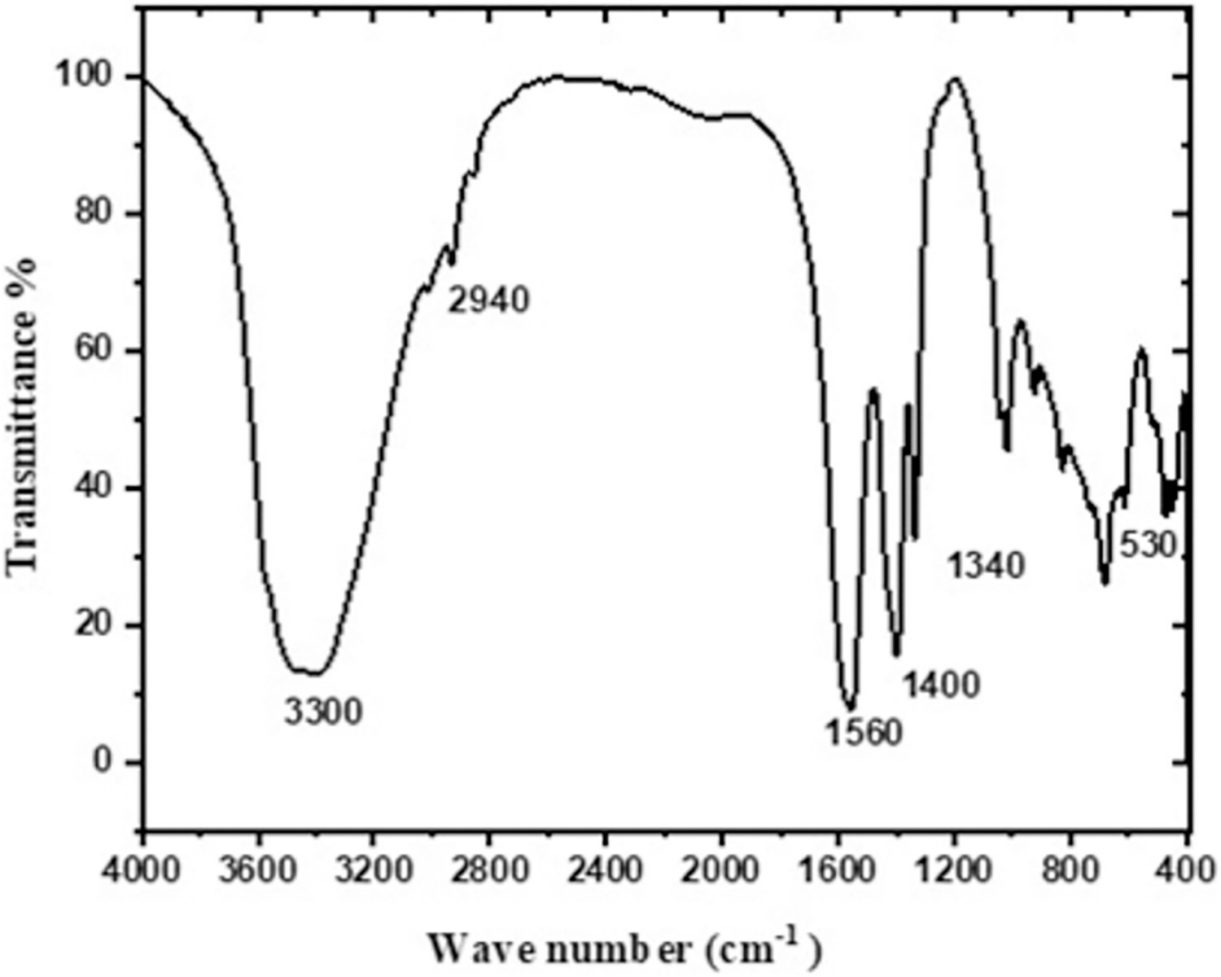
FT-IR spectrum of ZnO NPs prepared using ginger.

As shown in Fig 3, the IR spectrum of the produced formulations shows strong broad bands at 3300 and 2290 cm−1, an absorption bands at 1724, 1617, and 1449 cm^−1^ which indicate C = O stretching of carboxylic group. The characteristic band of the ZnO NPs stretching appeared at a range from 400 to 500 cm^−1^. These findings indicated the purity of the obtained ZnO NPs and are in agreement with previous studies [23].

### 2.3. X-Ray Diffraction (XRD) analysis of ZnO NPs

**Fig 4:**
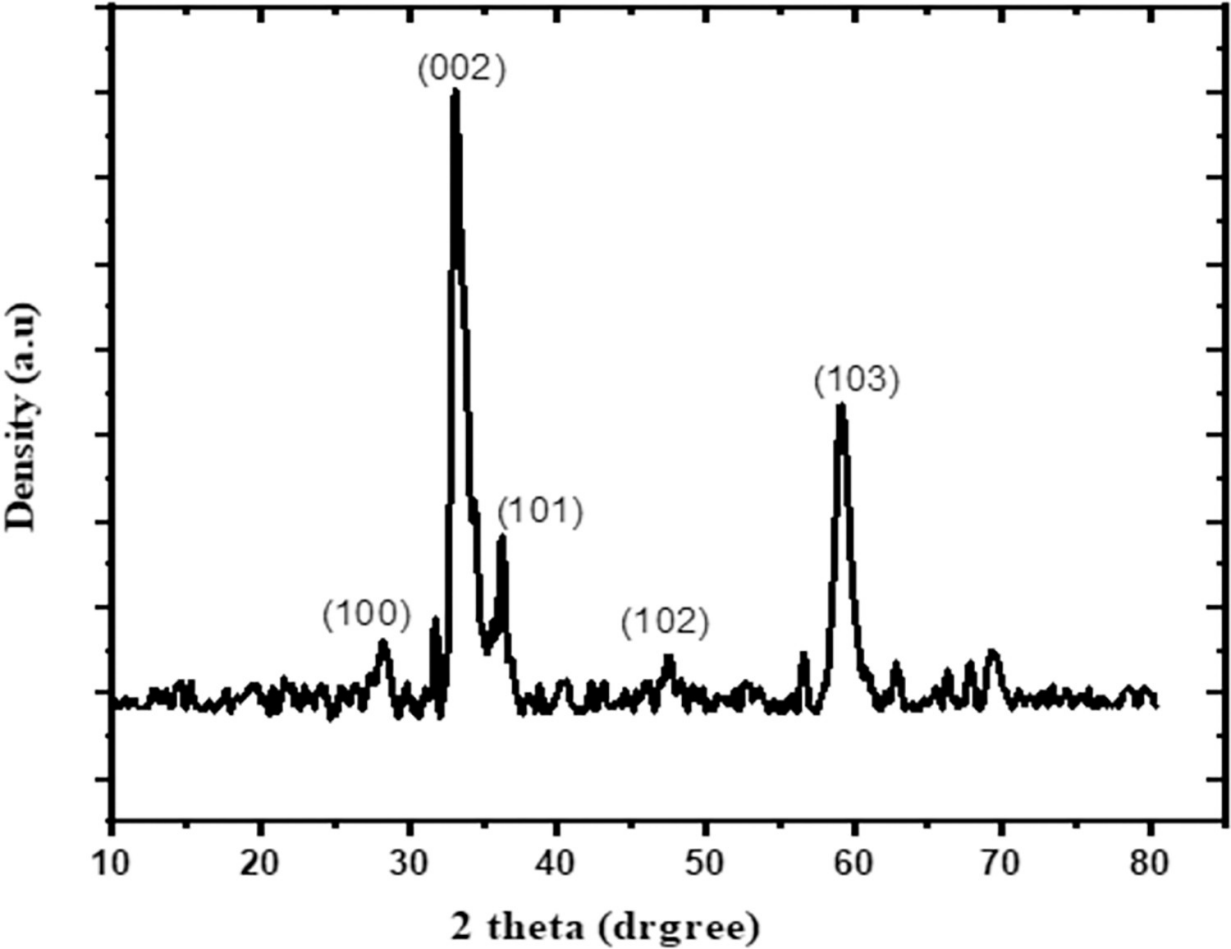
XRD pattern of the ZnO NPs prepared using ginger.

Purity and crystalline nature of ZnO NPs synthesized using ginger were ascertained by XRD analysis. The observed main distinct diffraction beaks at 2θ values were 31.71°, 33.34°, 36.25°, 47.54°, 62.76°, 68.00°, 69.10°, and 72.03° **(Fig 4)**. These beaks are parallel to 100, 002, 101, 102, 103, 200, 112 and 201, respectively planes of ZnO in the hexagonal Wurtzite structure corresponding with Joint Committee on Powder Diffraction Standards (JCPDS) (Card Number 36–1451). The average particle size calculated using XRD results was found to be 17.45 ± 2.26 nm (**Table 1**).

**Table 1:** Particle size of ZnO NPs calculated using XRD data.

Peak No.	2 θ	FWHM (β)	D (nm)	Average size (nm)
**1**	33.38698	1.25268	6.54702	17.45 ± 2.26
**2**	34.9853	0.82127	9.94341	
**3**	47.48163	0.67123	11.6765	
**4**	59.21791	1.16638	6.38223	
**5**	69.40634	0.77355	9.0997	

Abbreviation: FWHM (β): full width at half maximum of the diffraction peak, D is the calculated particle size in nm

### 2.4. Morphological examination of the synthesized formulation by Scanning Electron Microscopy (SEM)

**Fig 5:**
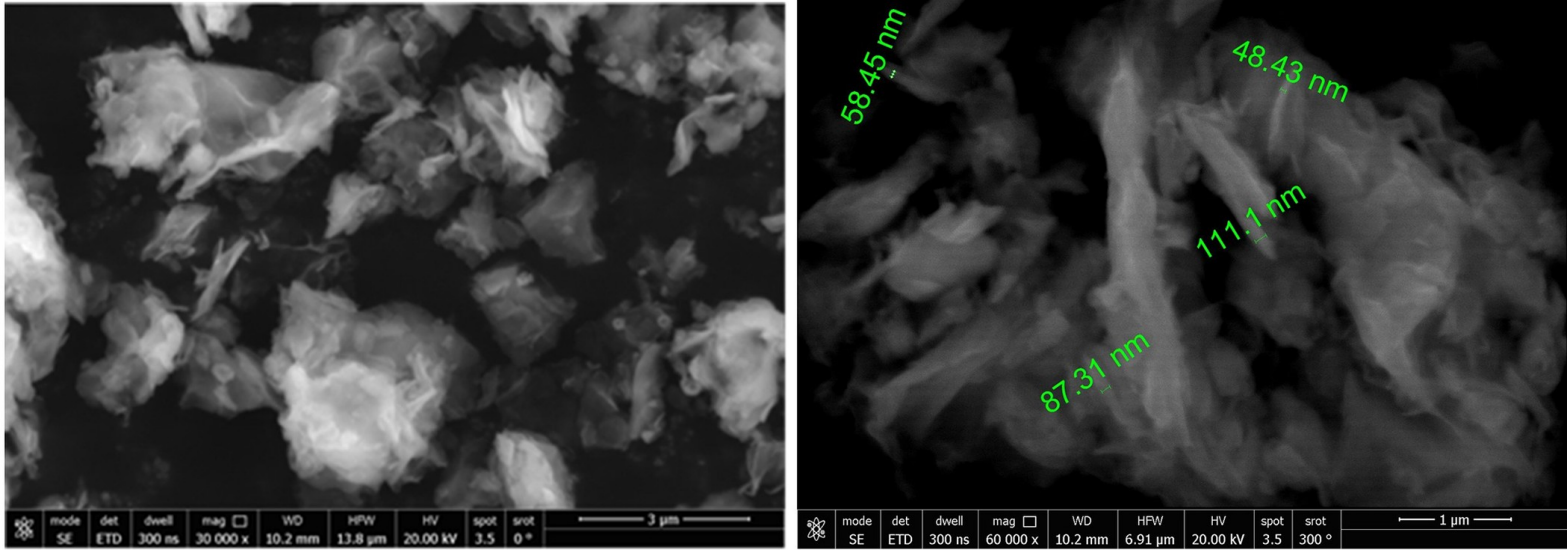
SEM images of ZnO NPs prepared using ginger showing particle size ranging from 43.3 to 111.1 nm (X 60,000).

SEM was used to study the morphology and size of the produced nanoparticles. The SEM image (Fig 5) shows the successful synthesis of ZnO NPs using the ethanolic extract of ginger as a reducing agent. The ZnO NPs were non spherical in shape with uniform distribution. The size of the obtained particles ranged from 43.3 to 111.1 nm.

## 3. Parasitological study

### 3.1. Survival time

**Fig 6:**
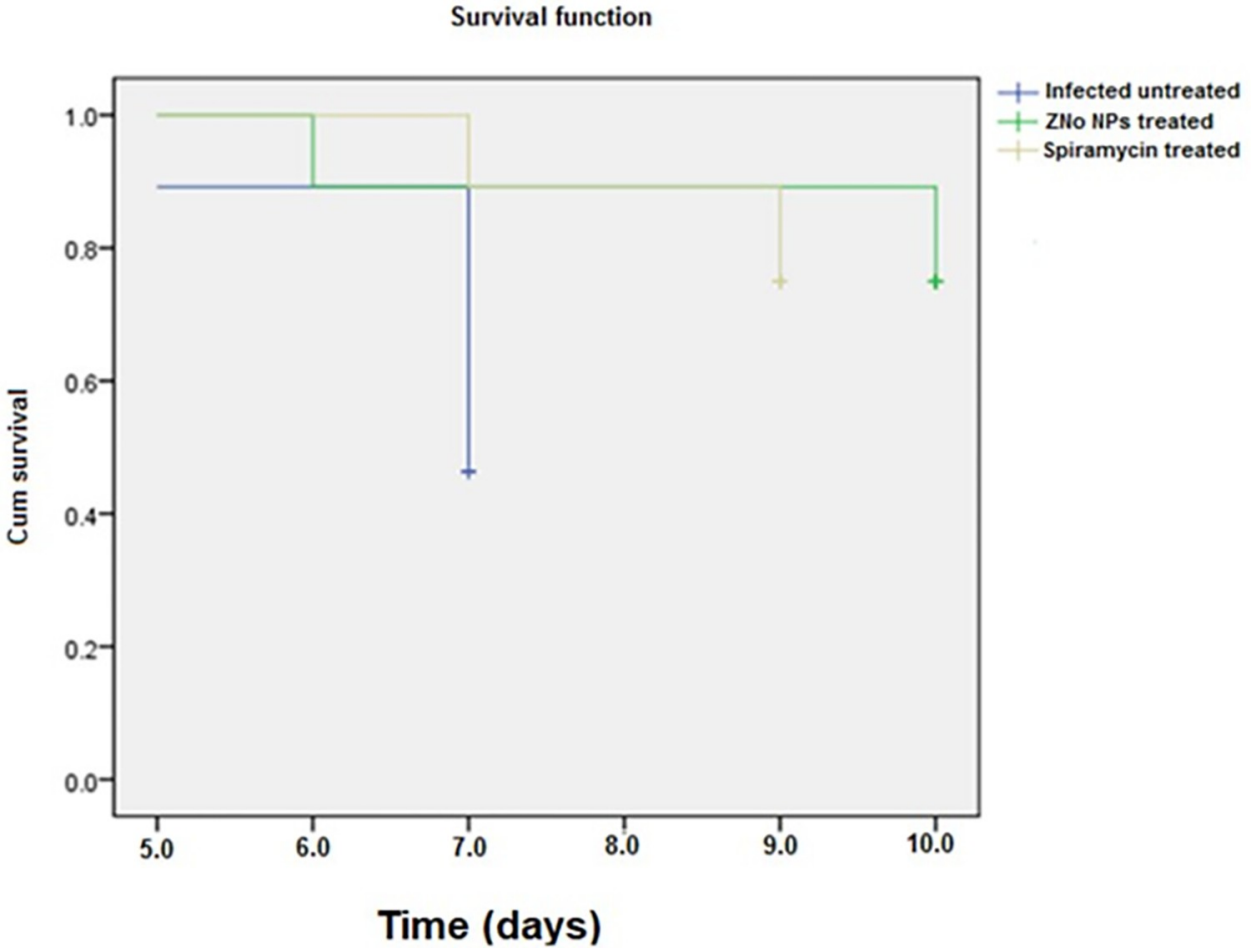
Kaplan-Meier overall survival curve for all groups showing the different survival time of untreated and treated animal groups. Animals treated with ZnO NPs have the longest survival time (10^th^ dpi) followed by animals treated with Spiramycin (9^th^ dpi).

The present study evaluated the anti-*Toxoplasma* efficacy of ZnO NPs synthesized on ginger in comparison to Spiramycin. Survival times for all animals were recorded. We recognized a significant difference in mice survival between treated and untreated subgroups (p< 0.05). On the fifth day post infection, infected untreated mice began to die, and none survived after the seventh day. In comparison to the untreated control group, the mean survival time was significantly longer in all treated groups. Mice treated with ZnO NPs had the longest survival times- where mice survived till the 10^th^ day post infection- while mice treated with Spiramycin had shorter survival times (9^th^ day post infection) (Fig 6).

### 3.2. Parasite load and percent reduction (%R)

The results of the present work showed that the treated and untreated groups had different parasite loads. Regarding liver impression smears, mice treated with Spiramycin or ZnO NPs showed a statistically significant reduction in the parasite load when compared to infected untreated mice. ZnO NPs therapy successfully reduced parasite load by 53.2%, producing greater reduction rate than animals treated with Spiramycin (48.1%) **(Table 2).**

**Table 2:** Mean tachyzoites count in liver impression smears and peritoneal fluid for all mice groups.

Animal group	Liver tachyzoites count Mean ± SD	*%R*	*P* value (among groups)	post hock test	Peritoneal fluid tachyzoites Mean ± SD	*%R*	*P* value (among groups)	Post hock test
Infected untreated	15.4 ± 3.1		0.002*		233 ± 231.9		0.095	
ZnO NPs treated group	7.2 ± 4.1	53.2		0.007 ^a^* 0.875 ^b^	11 ± 2.6	95.3		0.083^a^_,_ 0.324 ^b^
Spiramycin treated group	8 ± 3.03	48.1		0.003^a^*	67.2 ± 46.3	71.2		0.689^a^

***a*** letter indicates ***P*** value in comparison to infected untreated group

b letter indicates ***P*** value in comparison between ZnO NPs- and Spiramycin treated group using Tukey post hock test.

* indicates statistical significance.

On the same context, peritoneal fluid tachyzoites count in treated animals showed marked reduction than those of untreated group. ZnO NPs therapy successfully reduced parasite load by 95.3%, producing greater reduction rate than that produced by Spiramycin (71.2%) **(Table 2).**

### 3.3. Morphological examination of tachyzoites collected from different experimental groups by SEM

**Fig 7:**
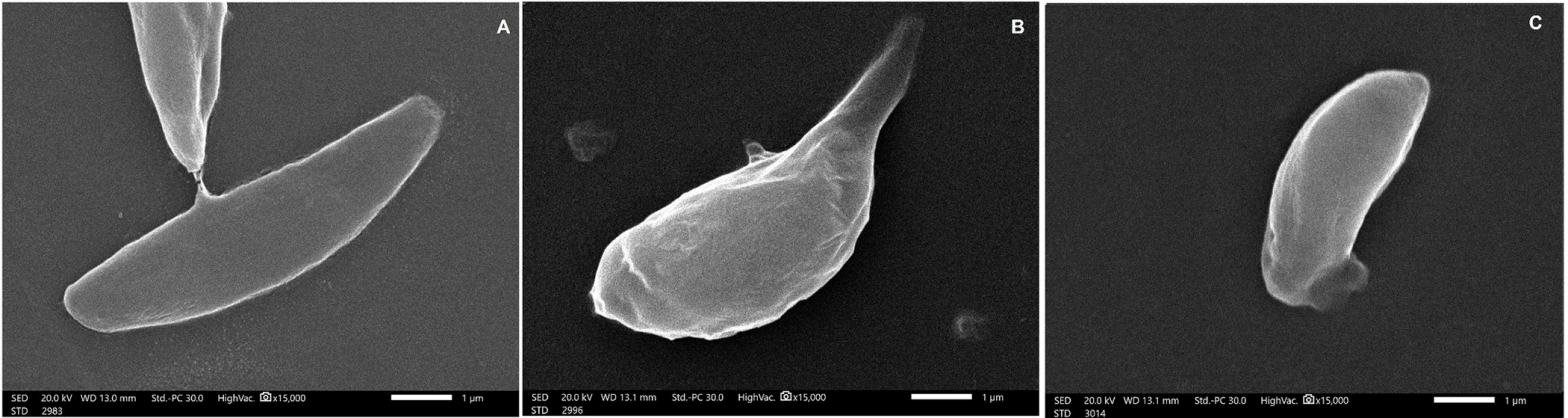
SEM examination of *Toxoplasma* tachyzoites. (A) Tachyzoites from infected untreated control, revealing crescent shape with apparent conoid (X 15,000). (B) Tachyzoites from ZnO NPs-treated group, showing shrunken tachyzoites losing normal crescent shape with evident loss of its smooth surface, erosion and protrusion of the surface, (X 15,000). (C) Tachyzoites from Spiramycin-treated group, showing shrunken organism losing its smooth conoid shape with furrows and ridges (X 15,000).

Tachyzoites from treated animals had markedly more distortion in the SEM than those from untreated animals. In the peritoneal fluid of infected untreated mice, tachyzoites were crescent-shaped -with one circular and one pointed pole- and smooth surfaces. The tachyzoites collected from treated groups, on the other hand, displayed pronounced deformation with loss of the smooth surface as well as erosions, protrusions, ulcerations, furrows, ridges, and/or blebs. Mice given ZnO NPs treatment showed more obvious morphological alterations (Fig 7).

## 4. Biochemical findings

### 4.1. liver functions

**Fig 8:**
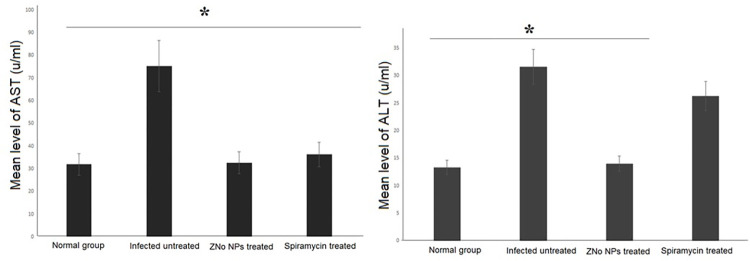
ZnO NPs treatment significantly improved liver enzymes in infected mice. Liver enzymes (AST and ALT) were measured in blood of normal, infected untreated, ZnO NPs treated and Spiramycin treated mice (10 mice/group). Data are expressed as means with error bars representing SD and were analyzed using ANOVA. Asterisk (*) indicates a significant difference in treated groups compared to the infected untreated group (p = 0.001).

Regarding AST level, untreated mice showed significantly higher AST level when compared to normal group (p = 0.001). Treatment of infected animals with either ZnO NPs or Spiramycin caused statistically significant reduction in AST level in comparison to the untreated animals (p = 0.003 and 0.002 respectively). Non statistically significant difference was reported between ZnO NPs- and Spiramycin -treated groups (p = 0.642), (Fig 8).

On the other hand, regarding ALT level, our findings showed significantly higher ALT level in untreated mice when compared to normal group (p = 0.001). Treatment of infected animals with ZnO NPs caused significant reduction in ALT level (p = 0.003) in comparison to the untreated animals. While treatment with Spiramycin caused non statistically significant difference of ALT level when compared to untreated animals (p = 0.201). In the present study, animals treated with ZnO NPs showed significantly lower levels of ALT in comparison to Spiramycin-treated animals (p = 0.049, Fig 8).

### 4.2. Effect of ZnO NPs on NO level and CAT activity

**Fig 9:**
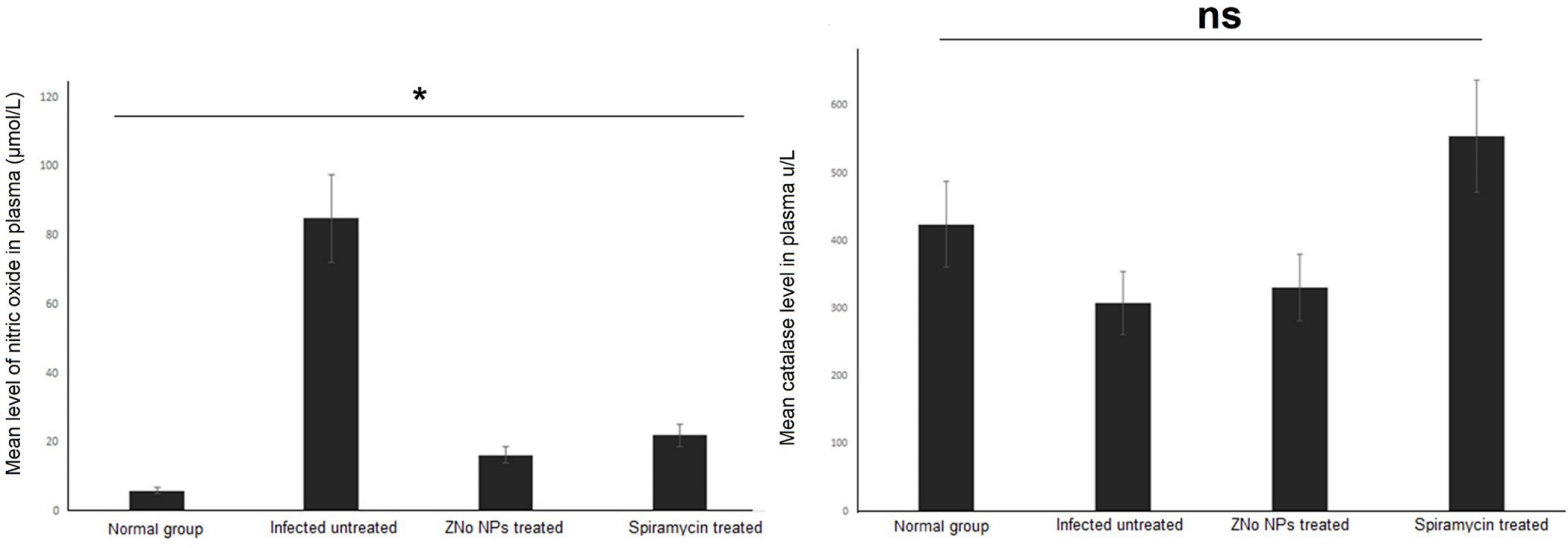
ZnO NPs treatment significantly reduced the oxidative stress as indicated by NO level and increased the antioxidant activity of CAT enzyme in infected mice. NO and CAT were measured in blood of normal, infected untreated, ZnO NPs- and Spiramycin-treated mice (10 mice/group). Data are expressed as means with error bars representing SD and were analyzed using ANOVA. Asterisk (*) indicates a significant difference in the mean level of NO in treated groups compared to the infected untreated group (p = 0.001), and “ns” indicates non-significant difference.

As shown in Fig 9, *T*. *gondii* infection resulted in a significant increase in the plasma levels of NO in infected non-treated group (p *=* 0.001). Treatment of infected animals with either ZnO NPs or Spiramycin caused statistically significant reduction in NO level in comparison to the untreated animals (p = 0.001 and 0.002 respectively). Lower mean of NO level was reported in ZnO NPs- than Spiramycin -treated group (with no statistical significance difference p = 0.119), (Fig 9).

Regarding CAT antioxidant enzyme level, Fig 9 shows low antioxidant enzyme activity in infected untreated animals when compared to normal control animals (with no statistical significance difference p = 0.655). Marked increase in the antioxidant enzyme activity was demonstrated in treated groups compared with the infected untreated group (with no statistical significance difference, p = 0.382 p = 0.286 for ZnO NPs- and Spiramycin- treated groups respectively).

## 5. Histopathological examination

### 5.1. Liver histopathology

**Fig 10:**
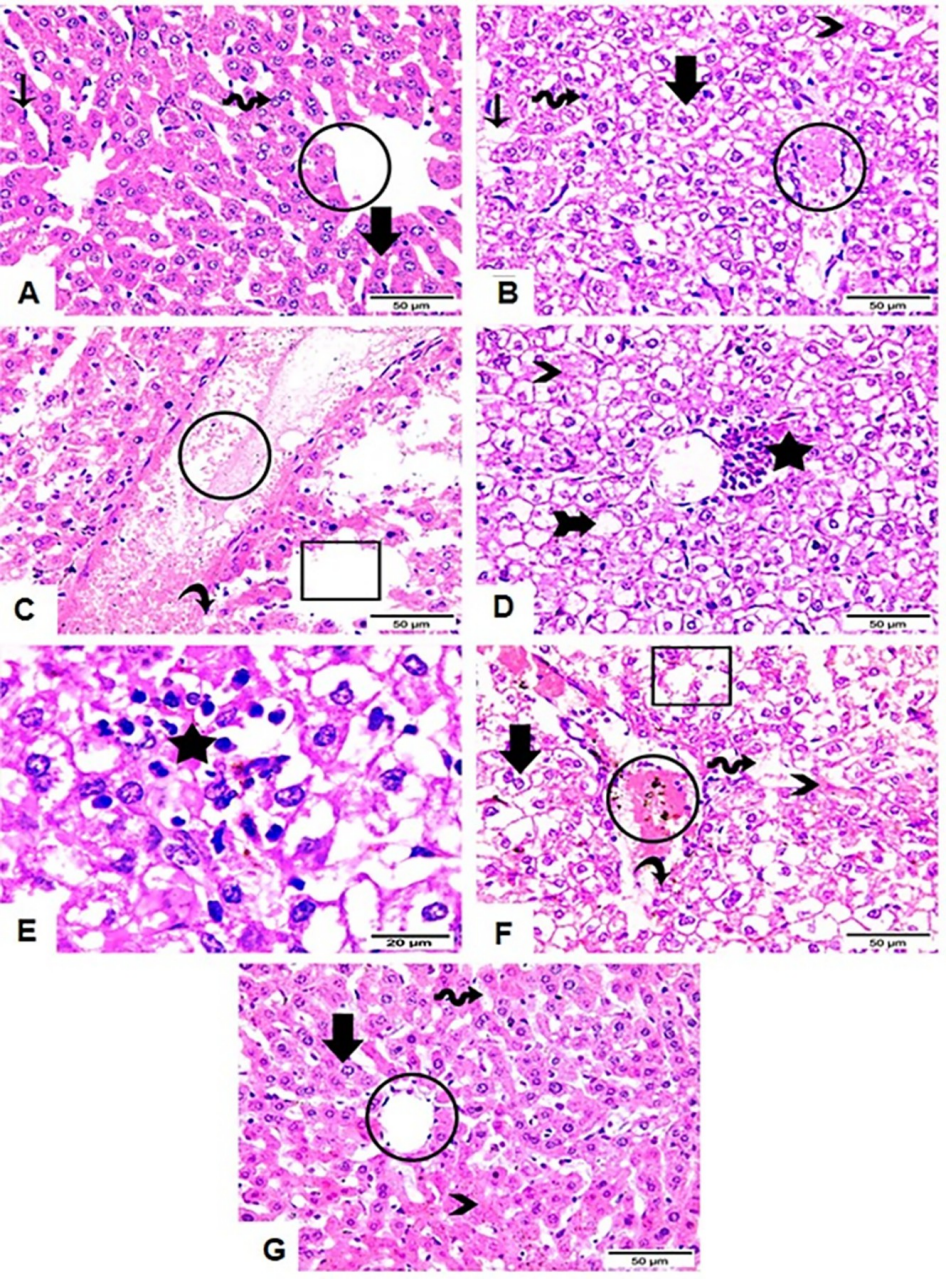
Photomicrograph of H & E stained liver tissue sections of all groups. (A) Liver section of normal mice showing normal central vein area with normal lining endothelium (circle); normal hepatic cords (thick arrow) consisting of polygonal hepatocytes with large round central light vesicular Uni or binuclear (wave arrow), and Kupffer cells (thin arrow) between hepatic cords and sinusoids. (B, C, D & E) Liver sections of infected untreated group showing serious degenerative and hydropic changes with loss of hepatic tissue normal assembly. The central vein showing severe congestion (circle) with deteriorated endothelium (curved arrow). Hepatocytes disclosed vacuolations (thick arrow), apoptotic nucleus (wave arrow) and lost nucleus (arrow with tail). Hepatic sinusoids showed obvious dilatation (thin arrow), necrotic changes (arrowheads) and edema leading to dispersion between hepatic cords (cube). Aggregated *T*. *gondii* tachyzoites are recognized (stars). (F) Liver section of Spiramycin treated group showing central vein congestion and activated macrophages with brown coloration (circle) and deteriorated endothelium (curved arrow). Edema (cube), necrosis (arrowhead), dilated hepatic sinusoids (wave arrow) as well as vacuolated hepatocytes (thick arrow) are still recognized. (G) Liver section of ZnO NPs—treated group demonstrated normal assembly of central vein (circle), hepatocytes (thick arrow), and hepatic sinusoids (wave arrow) with few necrotic foci (arrowhead).

Histopathological examination of normal control animals showed normal liver architecture. On the contrary, liver sections of infected untreated animals exhibited marked histopathological alterations with loss of hepatic tissue normal assembly. We demonstrated degenerative, necrotic and hydropic changes with congested central vein, dilated sinusoids, vacuolated apoptotic cells. *T*. *gondii* tachyzoites were also detected. Liver section of treated animals revealed restoration of normal liver tissue structure. Liver section of ZnO NPs treated animals showed much better liver structure with normal assembly of central vein, hepatocytes and hepatic sinusoids (Fig 10).

### 5.2. Histopathology of the brain

**Fig 11:**
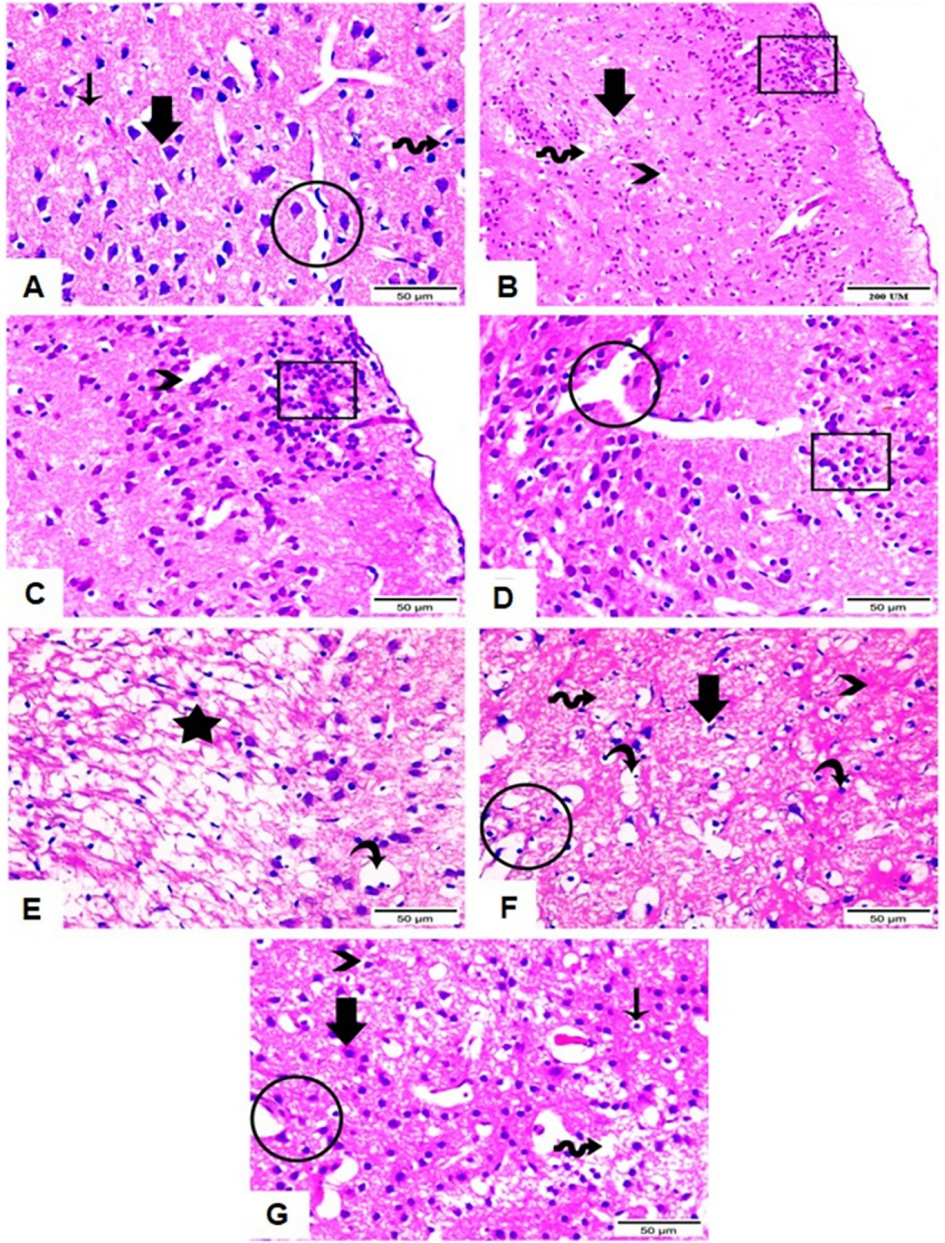
Photomicrograph of H & E stained brain tissue sections of all groups. (A) Brain section of normal group showing normal architecture of neuron (thick arrow), neuroglia cells (wave arrow), blood capillaries (circle) as well as neuropil (thin arrow). (B, C, D & E) Brain sections of infected untreated group showing severe degenerative changes with aggregated *T*. *gondii* tachyzoites under meninges (cube), neurons with vacuolated apoptotic nucleus (thick arrow) or lost nucleus (wave arrow), apoptotic and vacuolated neuroglia cells (curved arrow), dilated capillaries (circle), gliosis, edema leading to dispersion between neurofibrils (star), and vacuolations along neuropil (arrowhead). (F) Brain section of Spiramycin treated group showing apoptotic vacuolated neurons (thick arrow), apoptotic neuroglia cells (curved arrows), edema (wave arrow), necrotic areas (arrowhead), and dilated capillaries (circle). (G) Brain section of ZnO NPs treated group showing marked improvement with normal appearance of neurons (thick arrow) and blood capillaries (thick arrow). Neuroglia cells detected in regular form (arrowhead) and vacuolated apoptotic one (thin arrow). Edema and vacuolated neuropil (wave arrow) were still observed.

Microscopic examination of H & E stained brain section of normal animals demonstrated normal cortical architecture and structures. On the other hand, severe degenerative changes with dilated capillaries, gliosis and edema with aggregated *T*. *gondii* tachyzoites in brain tissue were demonstrated in infected untreated animals. Treatments with either ZnO NPs or Spiramycin induced brain structure improvement with normal appearance of neurons and blood capillaries (Fig 11).

## Discussion

*T*. *gondii* is one of the most common parasites in the world with a wide range of hosts. The parasite can cause serious disease in immunocompromised patients with limited treatment options owing to drugs side effects, high cost and emerging drug resistance [45]. These toxoplasmosis-related issues necessitate continued effort to look for new anti-toxoplasmosis medications. In particular, natural products continue to play a significant role in the search for medications in this category [46]. Using plants as reducing agents for green synthesis of NPs has drawn attention, because of their, eco-friendly, non-pathogenic, and cost-effective properties [47].

In the present study, we used ginger for green synthesis of ZnO NPs. Using UV-spectroscopy, green synthesized ZnO NPs showed a blue shift of an absorption peak at 290 nm confirming reduced particle size [48,49]. It is well documented that the size is a critical characteristic of metal nanoparticles that control their biological activities [49]. Smaller particle size leads to higher surface to volume ratio and easier penetration through the plasma membrane of the cell. Moreover, small sized nanoparticles are less toxic in vivo with better tissue distribution [24,25].

Our findings showed a good therapeutic potential for ZnO NPs in treatment of acute toxoplasmosis. A significant difference in survival time was reported between treated and untreated groups. Infected untreated mice, passes away rapidly within seven days PI due to aggressive tachyzoites dissemination in different organs with uncontrolled tachyzoites proliferation which is in agreement with previous experiments [33,36,37,39,50]. On the contrary, prolonged survival time observed in ZnO NPs treated animals (10 dpi) is an evidence of effective penetration of the drug to different tissues. This observation was supported by tachyzoites counts in peritoneal exudate and tissues which have both been found to be useful for evaluating the anti-*Toxoplasma* action of natural substances [51]. A significant reduction of the parasite burden in liver smears and peritoneal fluid of ZnO NPs treated animals when compared to the infected untreated group was recognized. This result is consistent with other studies that showed the high efficacy of nano-formulations in reducing the tachyzoites count in several organs in comparison to other drug formulation [39,52,53].

To further confirm our results, SEM–which allow high magnification in three dimensional perspectives—was done for the tachyzoites of treated and untreated groups [54]. The substantial deformity of tachyzoites collected from animals treated with ZnO NPs in contrast to untreated animals revealed high efficacy of the ZnO NPs treatment and effective tissue penetration. Similar results were noted after experimentally infected mice were treated with *Nigella sativa* oil, Aluvia, Nitazoxanide, Spiramycin, and Metronidazole [33,36,37]. The distorted tachyzoites morphology caused by ZnO NPs treatment prevent host cells invasion by the organism as manifested by reduced parasite load in the liver in the present study. These findings are supported by previous research [28,39,54,55]. To exert its effect on the tachyzoites structure, researchers concluded that the main target of ZnO NPs is the cytoplasmic membrane and DNA [37,43].

It has been postulated that high ALT and AST levels may be the early signs of *T*. *gondii* induced hepatic injury [56]. So, the reversal of these abnormalities by NPs provides an obvious evidence of improvement in hepatocyte functional status together with preservation of cellular architecture, demonstrating the hepato-protective effect of NPs [57]. The results obtained in the present work support this assumption. Our findings showed a statistically significant reduction in the level of ALT and AST in treated versus non treated animals. This finding was furthermore supported by the histopathological examination of the liver. Untreated infected mice exhibited severe hydropic and degenerative alterations as well as a loss of the normal assembly of the liver tissue. *T*. *gondii*-induced histological changes were improved by ZnO NPs therapy. Treatment of the infected animals restored the liver normal morphology, with a normal assembly of hepatocytes, sinusoids, and the central vein.

In the same context, ZnO NPs therapy improved the histopathological changes caused by *T*. *gondii* in the brain tissues. ZnO NPs were able to cure the severe degenerative brain alterations brought on by toxoplasmosis. The effectiveness of ZnO NPs indicate that the formulation has a great ability to infiltrate various tissues and pass the blood brain barrier [58,59].

One of the risk factors for causing oxidative injury and causing tissue damage is the overproduction of NO in response to the parasite infection. Additionally, it has been proposed that NO is a crucial component for the exit of parasites from non-immune cells and that it helped to create a persistent state of host-parasite equilibrium [60,61]. So in the present study, we evaluated the oxidant/antioxidant status in both treated and untreated animals. Our results demonstrated high levels of NO in infected untreated mice which significantly decreased after treatment with ZnO NPs. On the contrary. CAT antioxidant enzyme activity was markedly decreased in infected untreated animals in contrast to high levels in treated animals. The inhibition of antioxidant enzymes after toxoplasmosis is probably due to protein inactivation by ROS, as oxidative damage often leads to the loss of specific protein function [62]. Previous studies have revealed excellent antioxidant activity of ZnO NPs and high ability to suppress the production of iNOS [63]. These results are supported by results of the present study which showed high antioxidant activity and high CAT levels in agreement with previous studies [30,63,64].

### Conclusion

In this study, eco-friendly green synthesis of ZnO NPs was performed using ginger alcoholic extract as reducing agent. The produced formula showed a good therapeutic potential in treatment of murine toxoplasmosis as demonstrated by prolonged survival rate, reduced parasite burden, improved *T*. *gondii* associated liver injury (as shown by ALT and AST levels) and improved the oxidant/ antioxidant status in treated animals. ZnO NPs improved *T*. *gondii* induced brain and liver histopathological alterations indicating high ability of the formula to penetrate tissues and cross the BBB. Thus we assume that green synthesized ZnO NPs might be considered a possible new drug with a good therapeutic potential affording high levels of safety in the treatment of toxoplasmosis.
